# Epidemiology and multiple colonization of gastrointestinal pathogens in rural Tanzanian children with and without diarrhea: A case-control study

**DOI:** 10.1371/journal.pone.0305469

**Published:** 2024-06-18

**Authors:** Daniel Haile Chercos, Solomon T. Wafula, John P. A. Lusingu, Daniel T. R. Minja, Samwel Gesase, Joyce R. Mbwana, Ulrich Schotte, Jürgen May, Lea Mardeis, Anna Jaeger, Sandra Rojak, Maike Lamshöft, Joseph Kaseka, Eva Lorenz, Hagen Frickmann, Denise Dekker

**Affiliations:** 1 One Health Bacteriology Group, Bernhard Nocht Institute for Tropical Medicine, Hamburg, Germany; 2 Department of Infectious Disease Epidemiology, Bernhard Nocht Institute for Tropical Medicine, Hamburg, Germany; 3 National Institute for Medical Research, Tanga Centre, Tanga, Tanzania; 4 Department of Animal Health and Zoonoses, Central Institute of the Bundeswehr Medical Service, Kiel, Germany; 5 University Medical Centre Hamburg-Eppendorf (UKE), Tropical Medicine, Hamburg, Germany; 6 German Centre for Infection Research (DZIF), Hamburg-Lübeck-Borstel-Riems, Germany; 7 Department of Microbiology and Hospital Hygiene, Bundeswehr Central Hospital, Koblenz, Germany; 8 Institute of Medical Biostatistics, Epidemiology and Informatics, University Medical Centre of the Johannes Gutenberg University Mainz, Mainz, Germany; 9 Department of Microbiology and Hospital Hygiene, Bundeswehr Hospital, Hamburg, Germany; 10 Department of Medical Microbiology, Virology and Hygiene, University Medicine, Rostock, Germany; Universidad San Francisco de Quito, ECUADOR

## Abstract

Diarrheal diseases are important causes of morbidity and mortality, worldwide. The occurrence of multiple pathogens in stool samples of symptomatic and asymptomatic individuals in resource-limited countries have been repeatedly described. In this study, we assessed the differentiated effects of combined pathogen detections on recorded symptoms. A case-control study was conducted among 620 under-five-year-old children in rural northeastern Tanzania with emphasis of multiple detection. The median age of children was 11 months (IQR = 7, 20), and 52.1% were male. Cases (50.2%, n = 157) were less likely than controls (64.5%, n = 198) to have multiple colonization with gastrointestinal tract (GIT) pathogens. The children’s age was positively associated with the likelihood of harboring multiple GIT pathogens [OR, 1.02, 95% CI = 1.01, 1.04]. *Shigella* spp./enteroinvasive *Escherichia coli* (EIEC) [OR = 2.80, 95% CI 1.62, 4.83] and norovirus [OR = 2.04, 95% CI 1.23, 3.39] were more common in cases and were strongly associated with diarrhea, while enteroaggregative *E*. *coli* (EAEC) [OR = 0.23, 95%CI 0.17–0.33] were more common in controls. Diarrheal diseases in under-five children from rural Tanzania are likely to be due to infections with Shigella spp./EIEC, and norovirus with strongly age-dependent associations.

## Introduction

Diarrheal disease, particularly in children below the age of five, is among the leading causes of childhood mortality in resource-limited countries. In the year 2019 and thus before the SARS-CoV-2-pandemic-associated changes, approximately 1.6 million deaths were reported from diarrheal infections, mainly occurring in Africa and South-East Asia [1]. With few exemptions, however, microbial causes of infectious gastroenteritis are facultatively pathogenic, meaning that they can occur both as causative agents and as merely colonizing commensals in the enteric tract of human individuals. This is particularly true for tropical high endemicity, making assignments of etiological relevance to diagnostically detected microorganisms in stool samples challenging as summarized elsewhere [2]. However, reliable etiological assignments would be highly desirable to adequately consult the clinical management of patients with infectious gastroenteritis.

Large multicentric studies including the MAL-ED (malnutrition and enteric disease) study and others conducted in resource-limited countries have shown not only that the colonization with multiple gastroenteric pathogens is common in patients with diarrhea, but also demonstrated that multiple or individual pathogen species can be carried asymptomatically [1, 3, 4]. Asymptomatic infections are frequently overlooked, resulting in undetected but continued mild symptoms or colonization following a development of partial immunity. Recent studies among Tanzanian children with diarrhea not only showed that multiple detections of gastroenteric pathogens are frequent, but also that pathogens affect each other reciprocally regarding their etiological relevance in human disease [3, 4]. Furthermore, there seems to be an age-dependent increased likelihood of pathogen-specific infections in children below the age of five years [5]. In patients with infectious gastroenteritis, a broad spectrum of possible causative pathogens has to be considered including rare parasites as well as antimicrobial-resistant bacteria such as *Salmonella enterica* and others [6, 7]. Altogether, bacterial and protozoan pathogens quantitatively dominate in children with diarrhea in Tanzania.For instance among viral causative agents of gastroenteritis, rotavirus and norovirus have been most frequently implicated [8, 9]. Nevertheless, their role in causing symptoms and hence disease is often unclear, especially in patients from which multiple pathogens have been isolated. To identify associations between multiple pathogens and the occurrence of diarrhea, a control group without diarrhea would be essential.

The etiology of diarrhea in some regions of sub-Saharan Africa is still poorly understood, although crucial to guide antimicrobial treatment when indicated and to support the implementation of preventive programs.

This study aimed to assess the role of different gastrointestinal pathogens alone or in the course of multiple colonization in children with and without diarrhea attending a rural hospital in Tanzania. Further possible associations with cumulative effects of multiple infections/colonization were assessed as well as the most frequently observed pathogen combinations and gastrointestinal clinical symptoms in children aged below five years. By doing so, a new piece in the puzzle hopefully leading to a better future understanding of etiological relevance assignment to enteric microorganisms detected in tropical high-endemicity settings was provided.

## Material and methods

### Study design, population and sample materials

Unmatched case-control study was carried out from September 2017 to February 2019 among under-five years-old children at Korogwe District Hospital, in rural Northeastern Tanzania. Stool samples from 632 individuals with and without gastrointestinal symptoms were screened for bacterial, protozoan and viral pathogens with a focus on multiple pathogen detection. Symptomatic children presented to the outpatient department of Korogwe District Hospital were included in the study when they had diarrhea or a history of diarrhea in the past 72 hours (cases). Asymptomatic in the sense of the study approach were those children without diarrhea or a history of diarrhea in the past 72 hours before visiting the vaccinatinion clinic (controls). Clinical data such as vomiting and presence of diarrhea, as well as epidemiological data such as age, sex, and date of the year were also collected from the study participants. Cherry pit-sized stool volumes were collected and subjected to immediate nucleic acid extraction on site as well to storage at room temperature on Whatman FTA micro cards (Sigma Aldrich, Ulm, Germany, product number WHAWB120210) and on flocked swabs with an included proprietary drying system (GenoTube Livestock Swabs, Thermo Fisher Scientific Prionics AG, Schlieren, Switzerland) for later additional nucleic acid extraction after sample shipment to Germany for a methodical comparison reported elsewhere [10].

### Nucleic acid extraction

All stool samples were subjected to nucleic acid extraction by applying the Invisorb Spin Universal Kit (INVITEK Molecular, Berlin, Germany) for RNA and DNA according to the manufacturer’s instructions in the Korogwe GCLP-compliant laboratory, Tanzania. Extracts were then stored at -80°C and shipped on dry ice to the Bernhard Nocht Institute for Tropical Medicine (BNITM) in Hamburg, Germany after the Material Transfer Agreement was approved by the Tanzania National Ethics Committee. From a subpopulation of 466 patients with and without diarrhea, additional nucleic acid extraction was performed from Whatman FTA micro cards (Sigma Aldrich, Ulm, Germany, product number WHAWB120210) and from flocked swabs with an included proprietary drying system (GenoTube Livestock Swabs, Thermo Fisher Scientific Prionics AG, Schlieren, Switzerland) in Kronshagen, Germany. Fragments from the Whatman papers and swabs were incubated at 56°C in 550 μL buffer ATL (Qiagen, Hilden, Germany) and centrifuged for 15 minutes at 1000 rev min^-1^ (revolutions per minute) [11, 12]. Nucleic acid extraction was then performed automatically by applying a QIAsymphony automate (Qiagen; protocol “Complex_200_default_IC”, QIAsymphony DSP Virus/Pathogen Mini-Kit) as described by the manufacturer including the addition of carrier RNA. All extractions were stored at −80°C until further processing. In case of Polymerase Chain Reaction (PCR) assessments from multiple extractions from the same sample, the sample was considered as positive in case of at least one positive PCR signal from any extraction.

### Real-time PCRs

The samples were subjected to PCR-based screening in Hamburg and Kronshagen including in-house real-time multiplex PCR targeting enteropathogenic *Escherichia coli* (EPEC) (*EAF*, *eae*), enterotoxigenic *E*. *coli* (ETEC) (*LT /ST*), and enteroaggregative *E*. *coli* (EAEC) (*aggR*) [13], entero-invasive *Salmonella* spp. (*ttrR*), *Shigella* spp./enteroinvasive *E*. *coli* (EIEC) (*ipaH*), *Campylobacter jejuni (gyrA)*, and *Yersinia* spp. (*ail*) [14], the enteropathogenic protozoa *Entamoeba histolytica (SSU-rRNA)*, *Giardia duodenalis (SSU-rRNA)*, *Cyclospora* spp. (*SSU-rRNA*), and *Cryptosporidium parvum* (138-bp fragment inside the C. parvum-specific 452-bp fragment), the enteric helminths *Ancylostoma* spp. (*ITS-2*), *Ascaris lumbricoides (ITS-1)*, *Enterobius vermicularis (ITS-1)*, *Hymenolepis nana (ITS-1)*, *Necator americanus (ITS-2)*, *Schistosoma* spp. (*ITS-2)*, *Strongyloides stercoralis (18S-rRNA)*, *Taenia saginata (ITS-1)*, *Taenia solium (ITS-1)*, *Trichuris trichiura (18S-rRNA)* [15], and hepatitis E-virus [16], respectively, as well as one commercial real-time PCR targeting norovirus and hepatitis A virus (SureFast® Norovirus/Hepatitis A 3plex assay, r-biopharm, Darmstadt, Germany) according to the manufacturer’s instructions. The in-house real-time PCRs and their performance characteristics are described in detail elsewhere [13–17]. Both qualitative PCR results and cycle threshold (Ct) values were recorded.

### Statistical methods

Data were collected and managed using REDCap version 9.8.2 (Vanderbilt University, Nashville, USA) before being analyzed using R version 4.2.1 and RStudio version 2022.12.0+353 (Posit Software, Boston, Massachusetts). Categorical data were described as frequencies and percentages, and numerical data were summarized by calculating the median and interquartile range. The association between multiple colonization of gastrointestinal tract (GIT) pathogens and case-control status were estimated using ordinal logistic regression which is adjusted for sex, age and season. Binary logistic regression with interaction terms adjusted for sex, age, and season was also constructed to determine if there was a difference in the distribution of the most commonly reported combinations of gastrointestinal pathogens between cases and controls. Based on the p-value < 0.05 criterion, variables with significant associations were identified, and Benjamini and Hochberg correction was applied when multiple testing was conducted. Odds ratios (OR) with 95% confidence intervals (CI) were calculated to describe effect sizes.

## Results

### Demographic characteristics of study participants

Of the 632 children enrolled, complete information were available for 620 children (313 cases and 307 controls). Approximately half (52%, n = 324) were male and the median age was 11 months (IQR = 7, 20). Among recorded symptoms, vomiting was more prevalent in cases (38%) than in controls (11%) (q<0.001) (Table 1).

**Table 1:** Characteristics of children with diarrhea (cases) and without diarrhea (controls) (N = 620).

Characteristic	Overall, N = 620^1^	Cases, N = 313^1^	Controls, N = 307^1^	q-value^2^
**Sex**				0.349
Female	296 (48%)	144 (46%)	152 (50%)	
Male	324 (52%)	169(54%)	155 (50%)	
**Age groups (months)**				0.775
0–12	333 (54%)	169 (54%)	164 (53. 4%)	
13–24	181 (29%)	88(28%)	93 (30. 3%)	
Above 24	106 (17%)	56 (18%)	50 (16. 3%)	
**Vomiting symptom**				**<0.001**
Yes	151 (24. 4%)	118 (38%)	33 (11%)	
No	469 (75.6%)	195 (62%)	274 (89%)	

^1^n (%)

^2^Pearson’s Chi-squared test; Benjamini & Hochberg correction for multiple testing

### Common gastrointestinal pathogens isolated from rural Northeastern Tanzanian children

**Fig 1:**
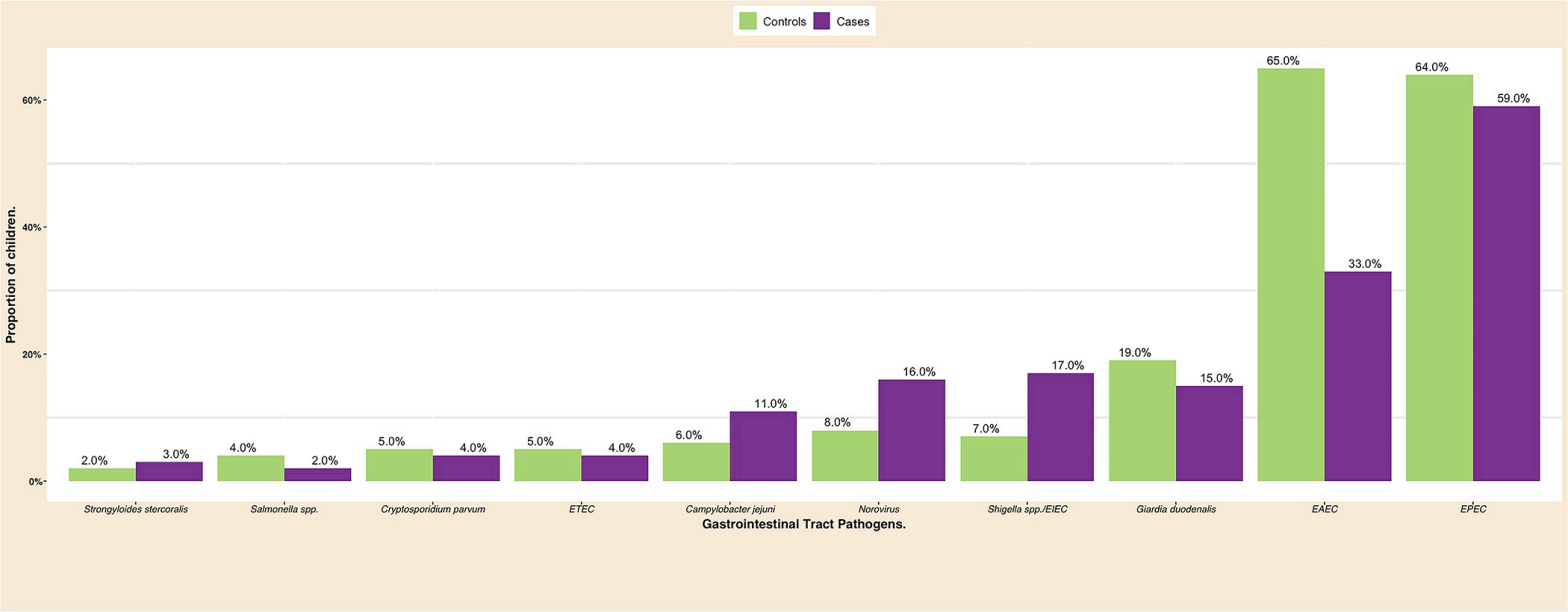
Ten most common GIT pathogens found in rural Northeastern Tanzanian children (N = 620).

Among the 23 gastrointestinal pathogens considered in this study, *G*. *duodenalis*, enteropathogenic *E*. *coli* (EPEC), and *enteroaggregative E*. *coli* (EAEC) were the most frequently observed. Despite their low proportions, *Shigella* spp./enteroinvasive *E*. *coli* (EIEC), norovirus, and *C*. *jejuni* were more common in cases than in controls. EAEC, EPEC, and *G*. *duodenalis* were observed more often in controls than in cases (Fig 1).

### Distribution of GIT pathogens in different age groups

**Fig 2:**
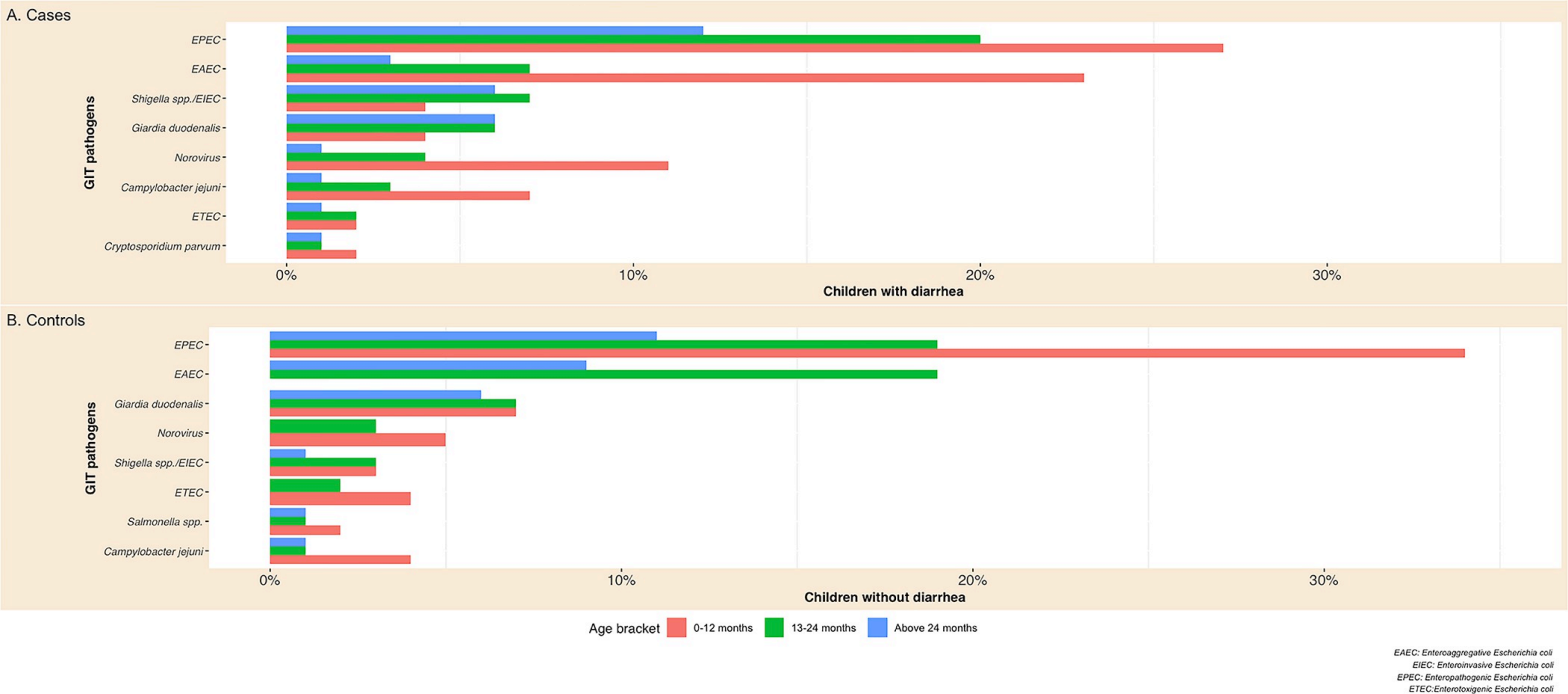
Distributions of common GIT pathogens among different age groups in rural Northeastern Tanzanian children (N = 620).

Enteropathogenic *E*. *coli* and EAEC were the most frequent GIT pathogens found in rural Northeastern Tanzanian children with and without diarrhea under the age of two. Norovirus was more frequent in children under the age of two in the case group than in children of comparable age in the control group. *C*. *jejuni* was more frequent in children with diarrhea under the age of two, compared to children of similar age in the control group. *Shigella spp*.*/*EIEC were more frequent in children above one year of age in cases than in children of similar age in the control group. The graph below (Fig 2) suggests that the frequency of any GIT pathogen decreases as children get older, with the exceptions of *Shigella* spp.*/*EIEC and *G*. *duodenalis*.

### Multiple GIT pathogens among rural Northeastern Tanzanian children

More than one GIT pathogen was isolated in 50.2% (n/N = 157/313) of cases and 64.5% (n/N = 198/307) of controls. On the other hand, more cases (33.5%, n/N = 105/313) than controls (25.4%, n/N = 78/307) had only a single GIT pathogen. More children tested negative for GIT pathogens (16.3%, n/N = 51/313) than the controls (10.1%, n/N = 31/307). Children with multiple colonization had up to five detected GIT pathogens. There were more controls than cases with two GIT pathogens (34.5%, n/N = 106/307 vs 29.1%, n/N = 91/313) and three GIT pathogens (23.4%, n/N = 72/307 vs 14.1%, n/N = 44/313). However, the proportion of children with four (5.55% n/N = 17/307 vs 5.54% n/N = 17/313) and five (0.98% n/N = 3/307 vs 1.6% n/N = 5/313) GIT pathogens in both groups was the same.

### Common GIT pathogen co-infection among rural Northeastern Tanzanian children

**Fig 3:**
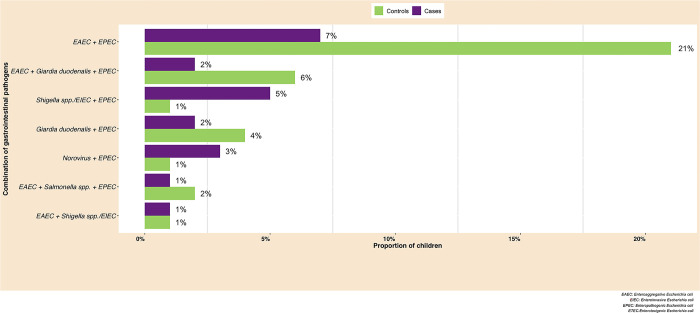
The most common gastrointestinal pathogen combinations observed in rural Northeastern Tanzanian children (N = 355).

The most common GIT combination found in rural northeastern Tanzanian children’s stool samples were EAEC and EPEC, which was more frequent in controls than in cases. In addition, the combined prevalence of EAEC, *G*. *duodenalis*, and EPEC in controls was more frequent than in cases (Fig 3). In contrast, the combination of *Shigella* spp./EIEC and EPEC was more frequent in cases than in controls.

### Associations of GIT colonization in rural Northeastern Tanzanian children

There was a difference in the odds of having multiple GIT pathogenic organisms when comparing cases to controls. Adjusted regression results revealed that cases were 44% less likely than controls to have multiple GIT pathogenic organisms isolated in their stool samples [OR = 0.56, 95% CI = 0.41, 0.76]. Similarly, there appears to be a minor but statistically significant association between children’s age and the odds of harboring multiple GIT pathogens. The odds of harboring multiple GIT pathogens increased by 2% on average for every additional 1 month of child age [OR, 1.02, 95%CI = 1.01, 1.04]. In contrast, there was no significant differences in odds of multiple colonizations between different gender (of the child) and season of the year (Table 2).

**Table 2:** Factors associated with multiple colonization of GIT pathogenic microorganisms in rural Northeastern Tanzanian children (N = 620).

	Colonization status			Regression			
				Unadjusted		Adjusted	
	No	Single	Multiple	OR^1^(95% CI^1^)	p-value	OR^1^(95% CI^1^)	p-value
**Gender**					0.11		0.12
Male	47 (15%)	101(31%)	176 (54%)	Ref		Ref	
Female	35 (12%)	82 (28%)	179 (60%)	1.28 (0.94, 1.75)		1.28(0.94, 1.76)	
**Group**					<0.001		**<0.001**
Controls	31 (10%)	78 (25%)	198 (64%)	Ref		Ref	
Cases	51 (16%)	105(34%)	157 (50%)	0.56(0.41, 0.76)		0.56(0.41, 0.76)	
**Season**					0.6		0.6
Dry Season	49 (14%)	101(29%)	195 (57%)	Ref		Ref	
Rainy Season*	33 (12%)	82 (30%)	160 (58%)	1.10(0.80, 1.49)		1.08(0.79, 1.48)	
**Age (months)**				1.02(1.01, 1.04)	0.005	1.02(1.01, 1.04)	**0.004**

^1^OR = Odds Ratio, CI = Confidence Interval

*January—May & November—December

### Association between GIT pathogens and diarrhea in rural Northeastern Tanzanian children

According to logistic regression analysis adjusted for age, gender and season, cases were 2.80 times as likely than controls to be associated with *Shigella* spp./EIEC infection [OR = 2.80, 95% CI 1.62, 4.83]. In addition, norovirus was twice as likely to be detected among cases compared to controls [OR = 2.04, 95% CI 1.23, 3.39]. Cases also had lower odds for EAEC detection than controls [OR = 0.23, 95%CI 0.17–0.33]. We observed a marginally significant association between *C*. *jejuni* infection and diarrhea (case group) [OR = 1.77, 95%CI 0.99–3.16] (Table 3).

**Table 3:** Distributions of GIT pathogens most commonly observed in rural Northeastern Tanzanian children with and without diarrhea (N = 620).

	Group		Regression		
Characteristic	Cases, N = 313^1^	Controls, N = 307^1^	aOR^2^,^3^	95% CI^2^	q-value^3^
*Strongyloides stercoralis*	8 (2.6%)	6 (2.0%)	1.34	0.45, 3.99	0.625
*Giardia duodenalis*	46 (15%)	60 (20%)	0.69	0.45, 1.07	0.100
*Campylobacter jejuni*	34 (11%)	20 (6.5%)	1.77	0.99, 3.16	**0.053**
*Cryptosporidium parvum*	11 (3.5%)	15 (4.9%)	0.70	0.31, 1.56	0.381
*Salmonella* spp.	7 (2.2%)	12 (3.9%)	0.54	0.21, 1.40	0.202
*Shigella* spp./EIEC^5^	53 (17%)	22 (7.2%)	2.80***	1.62, 4.83	**<0.001**
ETEC^6^	13 (4.2%)	17 (5.5%)	0.75	0.36, 1.59	0.458
EAEC^7^	101 (32%)	202 (66%)	0.23***	0.17, 0.33	**<0.001**
EPEC^8^	182 (58%)	199 (65%)	0.76	0.55, 1.05	0.096
Norovirus	49 (16%)	26 (8.5%)	2.04*	1.23, 3.39	**0.006**

^1^ n (%)

^2^ OR = Odds Ratios adjusted for age, sex and season of the year; CI = Confidence Interval

^3^ z-test

*q<0.05

**q<0.01

***q<0.001; Benjamini & Hochberg correction for multiple testing

^4^ EIEC: enteroinvasive *Escherichia coli*

^5^ ETEC: enterotoxigenic *Escherichia coli*

^6^ EAEC: enteroaggregative *Escherichia coli*

^7^ EPEC: enteropathogenic *Escherichia coli*

### The effect of GIT pathogens and their combinations on the presence of diarrhea

**Fig 4:**
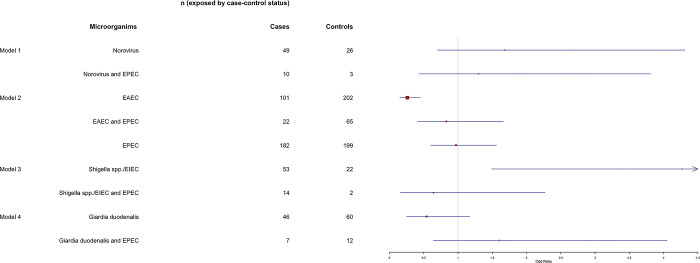
The effect of commonly observed multiple GIT pathogen colonization in case of the occurrence of diarrhea in rural Northeastern Tanzanian children (N = 620).

According to a logistic regression analysis that accounted for age, sex, and season, the odds of finding EAEC in cases were significantly lower compared to controls. Furthermore, the odds of identifying *Shigella spp*. /EIEC in cases were significantly greater than in controls. Furthermore, while there is no statistically significant difference between cases and controls, the presence of EPEC appears to influence the odds of identifying norovirus, EAEC, *Shigella spp*.*/EIEC*, and *Giardia duodenalis* in cases or controls (Fig 4).

## Discussion

The study sought to understand the epidemiology of gastrointestinal pathogens among under five children with and without diarrhea in rural Tanzania. The groups were comparable regarding age and gender, more vomiting in the diarrhea group compared to the control group further confirmed clinically apparent gastroenteritis within the diarrheal group. Our findings confirmed a high prevalence of bacterial and protozoan intestinal infections previously reported in Tanzanians [18, 19]. Among the identified enteropathogenic protozoa, *G*. *duodenalis* and–to a lesser extent–*C*. *parvum* were most frequent, which is in line with previous findings [20, 21]. *E*. *histolytica* did not play a relevant role in contrast to what was described before [22]. This observed discrepancy as compared to other studies [22] that were done elsewhere in Tanzania suggests geographic disparities in *E*. *histolytica* prevalence in Tanzania. Diagnostic accuracy of the screening PCR applied (mean and 95% confidence interval) of 97.7% (94.0%, 99.1%) sensitivity and 99.4% (98.7%, 99.7%) as reported elsewhere [23] makes it unlikely that relevant proportions of *E*. *histolytica*-containing samples were overlooked in the present study. Focusing on detected enteropathogenic bacteria, there was a great majority of diarrheagenic *E*. *coli* and *Shigella* spp. followed by *C*. *jejuni* and *Salmonella* spp. This finding is in line with previous reports in Tanzanian context [18, 19, 24], although two-digit percentages of *Salmonella* spp.-infections like reported elsewhere [24] could not be confirmed in our study. The quantitatively relevant detection of norovirus in our study cohort matches previous results [9]. Quantitatively relevant helminthic infections could only be shown for *S*. *stercoralis*, for which regional differences were previously demonstrated in another study conducted in Northern Tanzania [25].

Multiple pathogen detections were not primarily associated with increased risk of clinical diarrhea but were more frequently observed in colonized individuals than in patients with diarrhea. Moreover, multiple colonization was associated with age, while–in contrast—age-dependent pathogenicity was only inconsistently recorded. Although the numbers of older children were low, these findings at least pointed into the direction of habituation phenomena as recently described for Tanzania [26], potentially resulting in a semi-immunity-like state without clinically apparent diarrhea. This likely immunity-associated habituation effect is supported by the finding that proportions of most pathogen detections dramatically declined with increasing age in spite of an obvious high colonization pressure. Interestingly, *Shigella* spp./EIEC detections in diarrheagenic children seemed to be an exemption from this rule.

In addition, associations of pathogen detection with diarrhea could be confirmed for a minority of pathogens only, while most pathogens were almost evenly distributed among both study groups. *Shigella* spp. or enteroinvasive *E*. *coli*, not discriminable with the applied PCR assay, as well norovirus detections were found to be associated with diarrhea, while there was an association of enteroaggregative *E*. *coli* and children without gastroenteric symptoms. Both observations match previous findings from other settings [26, 27]. In a previous study in children in rural Ghana [28], *Shigella* spp./EIEC and norovirus were found to be more frequent in children with diarrhea compared to children without gastroenteric symptoms. In the same study, *Cryptosporidium* spp. and rotavirus were associated with diarrhea, the latter not examined and frequencies of *Cryptosporidium* spp. being low in this study, which makes it difficult to draw any conclusion. Focusing on the here-observed association of enteroaggregative *E*. *coli* and healthy individuals [29], a recent study on travelers’ diarrhea indicated the abundance of this microorganism in stool samples in general, considerably challenging its etiological relevance.

Of note, no conclusive information on the effect of pathogen combinations on etiological relevance could be obtained from this study. However, this may at least partly be due to the low sample size and so, future studies with a larger sample size would be required to draw definite conclusion from this.

The study has a number of limitations. First, the sample size is still fairly low, which makes any conclusions on rarely occurring pathogens difficult. Second, the applied real-time PCR assays comprise only a subset of microorganisms possibly associated with diarrhea in the study participants. Other pathogens such as rotavirus, which is strongly associated with diarrhea, were not screened. Accordingly, a lacking pathogen diagnosis does not necessarily mean the absence of an etiologically relevant microorganism not included in the real-time PCR panel. Finally, we abstained from including semi-quantification based on the cycle threshold values of real-time PCR in the assessments, which was decided for the following reasons. One reason is that Ct-value-based stratification would have made the compared populations too small and so, statistically meaningful results would have been unlikely. Also, Ct-value comparisons in a previous technical assessment with datasets from the same study population [10] had shown that Ct-values of individuals with and without diarrhea were virtually indistinguishable. Although complex pathogen interactions with microbial loads as a relevant influence cannot be excluded based on this previous experience, we feel justified to assume that semi-quantification is no reliable parameter by itself to assign etiological relevance to a detected microbial pathogen in the assessed population. For the assessment of complex interactions, however, future studies including higher sample sizes will be required.

## Conclusions

In the presented study on the epidemiology of diarrhea in rural Tanzanian children, multiple PCR detections of pathogens were more likely to occur in children without diarrhea than in those with diarrhea. Multiple pathogens detection was also associated with the children’s age. Diarrhea was mainly associated with *Shigella* spp./EIEC either alone or in combination with other pathogens like, e.g., norovirus, while, in contrast, enteroaggregative *E*. *coli* were more frequently detected alone or in defined combinations with other enteropathogens in subjects without diarrhea. The study results underline the difficulties of assigning etiological relevance to pathogen detections in stool samples in high endemic settings. Further research is desired to support the diagnostic discrimination of etiologically relevant pathogen detections from the identification of harmlessly colonizing bystanders not requiring therapeutic intervention under such circumstances.

## Supporting information

S1 Data Anonymized raw data used for this manuscript. (XLSX)
